# Integrated transgene and transcriptome reveal the molecular basis of *MdWRKY87* positively regulate adventitious rooting in apple rootstock

**DOI:** 10.3389/fpls.2023.1136616

**Published:** 2023-01-26

**Authors:** Qiuye Tian, Mengli Xu, Dongchen Wu, Chaoping Wang, Xianlin Wang, Qinqin Che, Zhengnan Li, Xiaozhao Xu

**Affiliations:** ^1^ College of Horticulture, Qingdao Agricultural University, Qingdao, China; ^2^ Engineering Laboratory of Genetic Improvement of Horticultural Crops of Shandong Province, Qingdao Agricultural University, Qingdao, China; ^3^ Laboratory of Quality & Safety Risk Assessment for Fruit (Qingdao), Ministry of Agriculture and Rural Affairs, Qingdao Agricultural University, Qingdao, China; ^4^ Shandong Academy of Grape, Shandong Academy of Agricultural Sciences, Jinan, China; ^5^ Weihai Yingjuval Nursery Limited Company, Weihai International Port Economic and Technological Develepment District, Weihai, Shandong, China; ^6^ College of Horticulture and Plant Protection, Inner Mongolia Agricultural University, Hohhot, China

**Keywords:** auxin, adventitious root formation, transcriptome, apple, rootstock

## Abstract

For most fruit and forest species vegetative propagated from elite genotypes, adventitious rooting is essential. The ability to form adventitious roots significantly decreased during the juvenile to adult phase change. Apart from the miR156-SPL pathway, whether there is another regulation mechanism controlling age-dependent adventitious rooting ability remained largely unknown. In the present study, we showed that *MdWRKY87* expression level was positively correlation with adventitious rooting ability. In addition, over-expressing of *MdWRKY87* in tobacco leads to enhanced adventitious rooting ability, more adventitious root number and accelerated adventitious rooting process. Comparative transcriptome profiling indicated that *MdWRKY87* overexpression can activate the expression of adventitious rooting-induced genes, such as *WOX11* and *AIL*. In addition, *MdWRKY87* overexpression can inhibit the transcription of adventitious rooting-repressed genes, such as *AUX/IAAs* and type-B cytokinin *RRs*. Collectively, here we demonstrated that higher expression level of *MdWRKY87* contributes to age-dependent adventitious rooting-competent in juvenile apple rootstock.

## 1 Introduction

The root system is able to adapt its architecture and morphology to the soil environment and physiological requirements of the plant in a flexible manner (Casimiro et al., 2003). Plant root systems typically consist of primary roots, lateral roots, and adventitious roots (Ji et al., 2022). Among these root types, ARs display high phenotypic plasticity in response to a variety of environmental stimuli (Li et al., 2022). Monocotyledons, like rice and maize, produce adventitious roots during normal development. In most tree species, especially poplars, cuttings are the primary means of propagation, and species differ in their ability to form adventitious root significantly. For most fruit and forest species propagated from elite genotypes, adventitious rooting is essential for proliferation. Plant tissue totipotency enables the adventitious root formation from non-root tissues, which has been extensively utilized for vegetative propagation of agricultural and forestry plants (Bellini et al., 2014). Most fruit species, such as apple rootstock, adventitious root formation is not easy to limit good varieties of asexual reproduction. Despite some preliminary studies on the mechanism of adventitious roots formation in difficult-to-root trees, and more in-depth research is needed.

In most tree species, the ability to form adventitious roots significantly decreased during the juvenile to adult phase change. Extensive research has been conducted to overcome problems related to the loss or reduction of the ability of difficult-to-root trees to form adventitious roots (Levy et al., 2014; Xu et al., 2017; Wang et al., 2019; Li et al., 2021). In our previous study, we have showed that the rooting rates of cuttings from juvenile and rejuvenated donor plants were significantly higher than those of cuttings from adult trees in *Malus xiaojinensis* (Xu et al., 2017). The high expression of miR156 is positively correlated with auxin-induced adventitious roots formation (Levy et al., 2014; Xu et al., 2017). MiR156 functions *via* its target gene *MxSPL26* in regulating adventitious root formation (Xu et al., 2017). Our previous research has indicated that *MxSPL26* inhibited *MxHB13* expression by directly binding to its promoter (Li et al., 2021). During the adult phase, MxSPL26 interacts with auxin-induced MxTIFY9 and co-represses *MxHB13* expression, leading to reduced AR formation (Li et al., 2021). Although several pathways and components of auxin-mediated molecular regulatory networks underlying adventitious root formation in apple have been identified, but the molecular mechanisms need to be investigated further.

The WRKY proteins are a superfamily of transcription factors found exclusively in plant (Rushton et al., 2010). The name of WRKY is derived from the highly conserved amino acid sequence containing WRKYGQK and the zinc finger-like motifs (Cys2-His2 or Cys2-HisCys) (Eulgem et al., 2000; Rushton et al., 2010). Based on both the number of WRKY domains and their zinc-finger motif, WRKYs can be divided into three distinct groups (Eulgem et al., 2000). The WRKYs have been found to function in seed dormancy, embryo and trichome formation, senescence, hormone synthesis, signal transduction, defense responses and abiotic stresses (Johnson et al., 2002; Lagace and Matton, 2004; Zhang et al., 2004; Li et al., 2015; Singh et al., 2017; Hu et al., 2018; Liang et al., 2020; Kang et al., 2021; Zhu et al., 2021). To date, only a small number of research have reported that WRKYs participate in adventitious roots formation. The group IIe *WRKY* gene of *Catalpa* Scop, *CbNN1* expression increased with increasing adventitious rooting ability (Wang et al., 2019). PuWRKY75, as a transcription activator, controls the low phosphorus driven adventitious root formation through up-regulating *PuLRP1* and *PuERF003* transcription in *Populus ussuriensis* (Wang et al., 2022). The functions of WRKYs in adventitious root formation remain to be investigated.

In this study, we identified a group IIe subfamily *WRKY* gene, *MdWRKY87* from apple rootstock. The *MdWRKY87* protein was found located in the nucleus and functions as a transcriptional repressor in both yeast and plant cells. Our results also indicated that *MdWRKY87* promoted adventitious rooting through regulating root-related gene involved in auxin signaling pathway.

## 2 Materials and methods

### 2.1 Plant materials

The leafy stem cuttings of *M. xiaojinensis* (Mx) was used as the materials, because Mx has a high apomictic rate to ensure the juvenile materials stability (Xu et al., 2017). Semi-lignified leafy cuttings (8-10 cm in length) were excised from basal suckers (juvenile phase, Mx-J) and shoots from the canopy of reproductively mature trees (adult phase, Mx-A). The bases of leafy cuttings were immersed 1~2 cm in depth into a 3.0 g L^-1^ indole butyric acid (IBA, Sigma-Aldrich, St. Louis, MO, USA) solution for 1 min (Xu et al., 2017). Cutting dipped in IBA-free water was used as a control. After plugging the cuttings into 50 cell trays containing fine sand, they were incubated in a solar greenhouse. The rooting ability was evaluated at 35 days after treatment. Three biological replicates, each with at least 50 leafy cuttings, were used for the experiment to manage experiment errors.

Tissue-cultured ‘M9T337’ plantlets were sub-cultured in Murashige and Skoog (MS) medium containing 7.5 g L^-1^ agar and 30 g L^-1^ sugar (pH 5.8) with 0.5 mg L^-1^ IBA and 0.2 mg L^-1^ 6-benzylaminopurine (6-BA) (Cheng et al., 2020). After 30 days, stem cuttings were transferred into 1/2 medium containing rooting 7.5 g L^-1^ agar and 30 g L^-1^ sugar (pH 5.8) with 0.5 mg L^-1^ IBA and 0.1 mg L^-1^ 1-naphthalene acetic acid (NAA) for rooting. The tobacco (*Nicotiana tabacum*) plants were sub-cultured in MS medium without hormone. The plantlets were grown under a 16 h light/8 h dark photoperiod with day/night temperatures of 25 ± 1°C and 20 ± 1°C.

### 2.2 Histological analysis

Paraffin sections of stem bases were prepared as previously described (Xu et al., 2017; Cheng et al., 2020; Jin et al., 2022), with some modifications. The bases of ‘M9T337’ stem cuttings were collected at 6, 9, and 12 days after transplanting on 1/2 MS medium with 0.5 mg L-1 IBA and 0.1 mg L-1 NAA. The bases of tobacco stem cuttings were excised at 2, 4 and 6 days after subculture on hormone-free MS medium. The samples were fixed in FAA solution (70% ethanol: formaldehyde: acetic acid, 95:5:5 [v/v/v]) for 2 days at room temperature, and store at 4 °C. Then samples were dehydrated with a graded ethanal series (50%, 70%, 85%, 95%, and 100%), infiltrated with xylene, and embedded in paraffin. Cross sections with a 10 μm in thickness were cut with a Leica RM2245 (Leica Microsystems, Wetzlar, Germany) rotary microtome, transferred onto glass slides, deparaffined with xylene, and re-hydrated through an ethanol series, and stained with toluidine blue. Slides were observed using an optical microscope DM2500 (Leica Microsystems, Wetzlar, Germany) and photos were obtained using an attached digital camera DFC420 (Leica Microsystems, Wetzlar, Germany).

### 2.3 Gene expression analysis

Total RNA was extracted from approximately 0.5 g of frozen sample using the TIANGEN Plant RNA Kit (TIANGEN biotech CO., LTD, Beijing, China, DP305). For each sample, 1 μg DNase-treated RNA was used to synthesize first-strand cDNA with oligo d(T) or random primer and HiScript^®^ II Q RT SuperMix (Cat. R223-01, Vazyme, China). A LightCycler 480 instrument (Roche, Basel, Switzerland) and ChamQ SYBR Color qPCR Master Mix (Vazyme, Nanjing, China) were used for qRT-PCR. The relative expression levels of genes were normalized to the reference gene *EF1α* and calculated using the 2 ^-ΔΔCt^ method (Livak and Schmittgen, 2001). All reactions were performed with at least three biological replicates. The primers are listed in **Supplemental Table S1**.

### 2.4 Histochemical GUS staining

The *MdWRKY87* promoter fragment (-2000 bp to -0 bp from the *MdWRKY87* ATG start codon) was inserted into pCambia1391 vector generating the *proMdWRKY87: GUS* construct. Tobacco leaves were transformed with the *Agrobacterium tumefaciens* strain GV3101 cells harboring a *proMdWRKY87: GUS* or *DR5: GUS* construct. Agrobacterium cells were re-suspended in buffer with (10 mM MgCl_2_, 10 mM MES-KOH, pH 5.6; adding 200 μM acetosyringone immediately prior to use) to an OD_600_ of 0.8~1.0. After injected with 1 ml needleless syringes, the leaves were treated with 50μM IBA and collected 6 hours later. The leaves were submerged in the GUS staining solution for 24 h at 37°C. After staining, tissues were cleared by immersing in 70% ethanol. All primers used are listed in **Supplemental Table S1**.

### 2.5 Subcellular localization

The ORF fragment (stop codon removed) of *MdWRKY87* containing *Sma*I and *Xba*I sites were inserted into the Super1300-GFP vector to generate the *pSuper : MdWRKY87*-*GFP* construct. Subcellular localization was conducted as previously described (Cheng et al., 2021). *A. tumefaciens* cells (GV3101) expressing *pSuper : MdWRKY87-GFP* and a Cherry-labelled nuclear marker (NF-YA4-mCherry) was re-suspended using the buffer (10 mM MgCl2, 10 mM MES-KOH, pH 5.6; 200 μM acetosyringone). *pSuper : NF-YA4-mCherry* was used as a nuclear marker. The tobacco leaves were injected with the re-suspended *A. tumefaciens* cells using a 1 ml needleless syringe. Three days after infiltration, fluorescence signals of the infiltrated leaves were detected using a laser scanning confocal microscope (Leica TCS SP5 II, Wetzlar, Germany). The primers used for construction are listed in **Supplemental Table S1**.

### 2.6 Transcriptional activation analysis in yeast

The coding fragment of *MdWRKY87* were fused to the GAL4-BD in pBD-GAL4 vector. The transcriptional activation analysis was conducted as previously described (Cheng et al., 2021). The transactivation activity was verified by the growth of yeast AH109 harboring full-length of MdWRKY87 on SD/-Trp and SD/-Trp-His plates and was confirmed by a X-α-Gal staining assay. All primers used are listed in **Supplemental Table S1**.

### 2.7 Transcriptional activation analysis in *N. benthamiana*

The *MdWRKY87* ORF sequence without stop codon was cloned into the pBD-VP16 vector (Han et al., 2016). The reporter vector contained a GAL4-luciferase (LUC) containing five copies of the GAL4-binding element and a minimal CaMV35S promoter at the 5’ end of the LUC gene (Han et al., 2016).The effector vectors or reporter vectors were introduced into *A. tumefaciens* strain GV3101. The *A. tumefaciens* cells was re-suspended to an OD_600_ of 1.0 using the buffer (10 mM MgCl_2_, 10 mM MES-KOH, pH 5.6; 200 μM acetosyringone). *A. tumefaciens* cells harboring effector vector and reporter vector were mixed 1:1, then injected into the tobacco (*N. benthamiana*) leaves by using a 1 mL needleless syringe. After spraying 1 mM luciferin onto the leaves, luciferase imaging was performed using NEWTON 7.0 (VILBER LOURMAT, Paris, France). An assessment of LUC and REN activities was conducted using the Duo-Lite Luciferase Assay System (DD1205–01, Vazyme, Nanjing, China) and BioStack Ready (BioTek Instruments Inc., Winooski, Vermont, USA). LUC/REN ratio was used to calculate the results. The primers used for construction are listed in **Supplemental Table S1**.

### 2.8 Transgenic tobacco generation

The *MdWRKY87* ORF sequence without stop codon was cloned into pRI101 vector to generate *35S*:*MdWRKY87-OE* construct. The construct was introduced into wild type tobacco (*N. tabacum*) leaves by *A. tumefaciens*-mediated transformation as previously described (Zhu et al., 2022). The infected leaves were selected on MS medium containing 100 mg L^-1^ kanamycin and 300 mg L^-1^ cefotaxime sodium to generate *MdWRKY87*-overexpressing (*MdWRKY87*-OE) transgenic lines. Transgenic plants were propagated by subculture on hormone-free MS medium. The primers used for construction are listed in **Supplemental Table S1**.

### 2.9 RNA-Seq

Total RNA was extracted from the stem bases of MdWRKY87-OE and wild type plants using the TIANGEN Plant RNA Kit (TIANGEN biotech CO., LTD, Beijing, China, DP305). A total amount of 3 µg RNA per sample was used in RNA-seq library construction. An Illumina Hiseq (Illumina, CA, USA) system was used for RNA sequencing by Novogene (Novogene, Tianjin, China). A quality assessment was performed on raw data using FastQC. Following Trimmomatic filtering out adapters and unpaired reads, the remaining clean reads were used to calculate the expression of gene by using Kallisto, an RNA-seq quantification program (Bray et al., 2016). The N. tabacum genome was used as the reference genome (Edwards et al., 2017). The count of reads was normalized to Transcripts Per kilobase of exon model per Million mapped reads (TPM). The log2TPM values were subjected to generate the heat map by TBtools software (Chen et al., 2020). The RNA-seq data were deposited in the NCBI Sequence Read Archive (accession number PRJNA917351).

## 3 Results

### 3.1 *MdWRKY87* expression correlates positively with adventitious rooting

**Figure 1:**
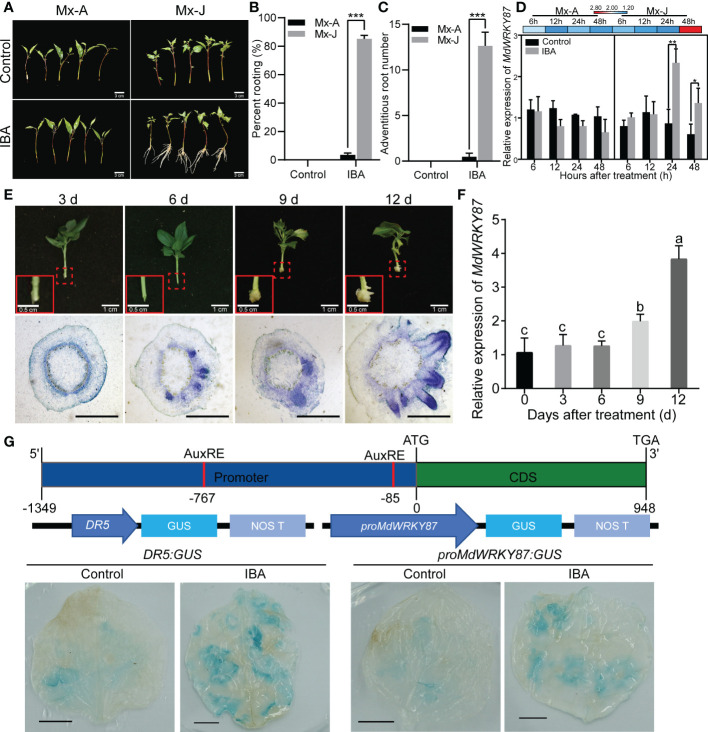
The expression of *MdWRKY87* is positively correlated with auxin-induced adventitious root formation. **(A)** Indole butyric acid (IBA) treatment induced adventitious root formation of leafy cutting from juvenile (Mx-J) not adult (Mx-A) phase in *M. xiaojinensis*. Scale Bar = 3 cm. **(B)** Adventitious rooting percentage of leafy cutting from Mx-A and Mx-J phase of *M. xiaojinensis* after IBA treatment. **(C)** Number of adventitious roots. For B and C, the mean values ± SD are shown for three biological replicates. Student’s *t*-test, *** *P* < 0.001. **(D)** Relative expression of *MdWRKY87* gene in leafy cutting of Mx-A and Mx-J after IBA treatment. The mean values ± SD are shown for three biological replicates. Stem bark samples of 0.5 to 1.0 cm basal sections of 20 Mx-J or Mx-A cuttings treated with IBA-free solution or IBA were pooled together as one biological replicates. Student’s *t*-test, ** *P* < 0.01. **(E)** The process of adventitious root formation of tissue culture plantlets growing on 1/2 MS medium containing IBA. Top, representative pictures of adventitious root formation of plantlets. The red dotted boxes indicate portion of stem base magnified in red solid line boxes (Scale bar = 0.5 cm). Scale bar = 1 cm. Bottom, transverse sections of stem base during the adventitious root formation. Scale bar = 1 mm. **(F)** Relative expression of *MdWRKY87* gene in stem base of tissue culture plantlets. Different letters indicate statistically significant differences (*P* < 0.05) at by Duncan’s test. **(G)** GUS activity of *N. benthamiana* leaves transiently transformed with *proMdWRKY87:GUS* or *DR5:GUS* after IBA treatment. Top, schematic representation of *MdWRKY87*. Medium, schematic representation of the *proMdWRKY87:GUS* and *DR5:GUS* constructs. Bottom, GUS activity analysis. Scale bar = 1 cm. *p < 0.05.

According to our previous research, semi-lignified leafy cuttings from Mx-J and shoots from the canopy of Mx-A were used in this study (Xu et al., 2017; Li et al., 2021). As the previous results (Xu et al., 2017; Li et al., 2021), Mx-J cuttings exhibited a high adventitious rooting ability (**Figures 1A–C**). After IBA treatment, the rooting percentage of Mx-J cuttings (85.14%) was significantly higher than that of Mx-A cuttings (3.57%, **Figure 1B**). Neither the cuttings of Mx-A nor Mx-J exhibited the ability of adventitious rooting (**Figure 1B**). Moreover, the adventitious root number per cutting of Mx-J was significantly more than that of Mx-A (**Figure 1C**). We previously identified the expression of the WRKY transcription factor family genes in the cutting stems of Mx-A and Mx-J after IBA treatment (Che et al., 2021). The expression of *MdWRKY87* gene was significantly induced in the Mx-J cutting treated with IBA (**Figure 1C**). However, there was no difference in the mRNA levels of *MdWRKY87* in the Mx-A cuttings treated with IBA or untreated control (**Figure 1C**).

To further define the relationship between *MdWRKY87* expression levels and adventitious rooting formation, we next examined the expression pattern of *MdWRKY87* during adventitious rooting formation of tissue culture plantlets in apple. According to the paraffin sections of stem bases of apple plantlets, we found that primordia with dome-shaped adventitious structures were clearly visible at 6~9 days after transplanting (**Figure 1E**) as our previous results (Cheng et al., 2020). After 12 days of transplantation, adventitious roots began to appear. As expected, the gene expression levels of *MdWRKY87* increased significantly with the emergence of adventitious root from stem base (**Figure 1F**).

To further validate *MdWRKY87* expression in response to IBA treatment, *Agrobacterium tumefaciens* cells (GV3101) harboring the *proMdWRKY87:GUS* or *DR5:GUS* construct (auxin-responsive reporter) were transiently transformed into tobacco leaves. After IBA treatment, the levels of GUS proteins obviously increased in both of leaves transformed with *proMdWRKY87:GUS* and *DR5:GUS* construct (**Figure 1G**), suggesting that the promoter activity of *MdWRKY87* responds to auxin. These results indicate high *MdWRKY87* expression correlates positively with adventitious rooting and may regulate auxin-mediated adventitious root development.

### 3.2 *MdWRKY87* is located in the nucleus and functions as a transcriptional repressor

**Figure 2:**
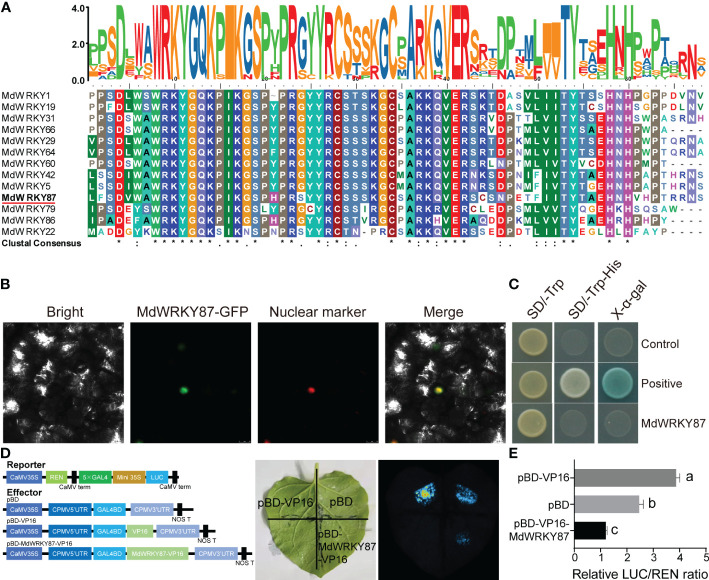
MdWRKY87 is a transcription repressor of WRKY family. **(A)** Sequence comparison of WRKY domains from WRKY IIe proteins, shaded by conserved amino acids. The accession numbers of all genes used are listed in the Methods section. **(B)** Subcellular localization of MdWRKY87 in *N. benthamiana* leaves. *Agrobacterium* carrying the pSuper : *MdWRKY87-GFP* and pSuper : *NF-YA4-mCherry* as a nuclear marker genes was co-infiltrated into *N. benthamiana* leaves. Images were captured 2 d following agroinfiltration. **(C)** Transcriptional activity analysis of MdWRKY87 protein in yeast. The transactivation activity was verified by the growth of yeast AH109 harboring *MdWRKY87* ORF on SD/-Trp and SD/-Trp-His plates and was confirmed by a X-α-Gal staining assay. **(D)** Transcriptional activity analysis of MdWRKY87 in *N. benthamiana* leaves. Reporter and effector constructs were co-infiltrated into *N. benthamiana* leaves. Left, schematic representation of the effector and reporter constructs. Right, live imaging of *N. benthamiana* leaves expressing reporter and effector constructs. **(E)** The dual-luciferase activity assay. The values were determined by calculating the ratio of LUC activity to REN activity (LUC/REN). Different letters indicate statistically significant differences (*P* < 0.05) at by Duncan’s test.

Multiple sequence alignments of WRKY22 homologs from apple indicated that *MdWRKY87* harbored a conserved WRKYGQ domain and a C2H2 (C-X5-C-X23-H-X1-H) zinc-finger motif at its C terminus (**Figure 2A**) and belonged to WRKY group IIe (Eulgem et al., 2000). To confirm whether *MdWRKY87* functions as a transcription factor, we expressed *MdWRKY87* fused to green fluorescence protein in tobacco leaves and observed that the fusion protein localized to the nucleus (**Figure 2B**). A transactivation assay in yeast indicated that the *MdWRKY87* protein has transcriptional activation activity (**Figure 2C**). We also performed a dual-luciferase transactivation assay in tobacco leaves. The results showed that the luciferase activity of co-expression of the reporter with pBD- WRKY87 -VP16 was significantly lower than that in the pBD-VP16 control (**Figures 2D, E**). These results suggested that *MdWRKY87* is indeed a transcription repressor of WRKY group IIe subfamily.

### 3.3 *MdWRKY87* positively regulates adventitious root development in tobacco

**Figure 3:**
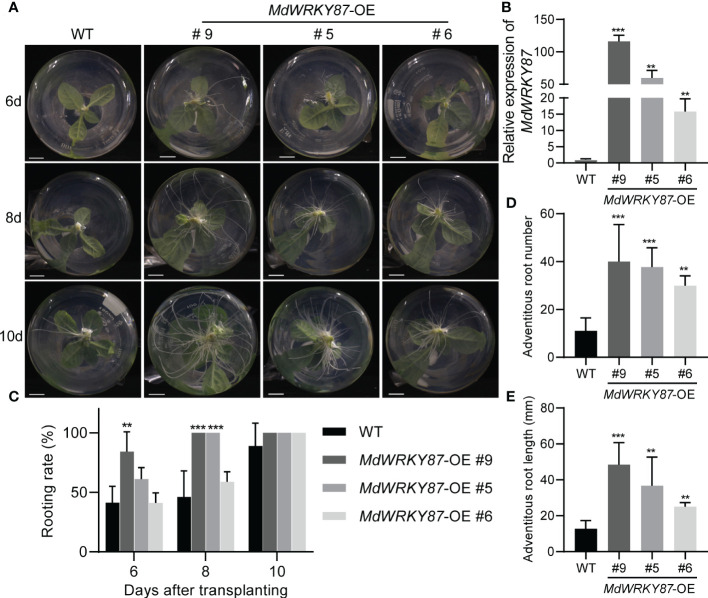
*MdWRKY87* accelerates adventitious root formation in transgenic tobacco (*N. tabacum*) plants. **(A)** Phenotypes of adventitious roots in wild-type (WT) and independent transgenic lines (*MdWRKY87-*OE9, *MdWRKY87-*OE5, and *MdWRKY87-*OE6) in the absence of auxin at 6, 8 and 10 days after transplanting into hormone-free MS medium. **(B)** qRT-PCR analysis of *MdWRKY87* expression in WT and *MdWRKY87-*OE lines. **(C)** Adventitious rooting percentage of wild-type (WT) and independent transgenic lines at 6, 8 and 10 days after transplanting into hormone-free MS medium. **(D, E)** Number **(D)** and length **(E)** of adventitious roots at 10 days after transplanting into hormone-free MS medium. The mean values ± SD are shown for three biological replicates. Asterisks indicate significant differences between WT and each transgenic lines by Student’s *t*-test (**P< 0.01; ***P< 0.001).

To characterize the role of *MdWRKY87* in adventitious rooting, we generated overexpression lines of *MdWRKY87* (*MdWRKY87*-OE) in tobacco plants by *Agrobacterium*-mediated transformation (**Supplemental Figure S1**). We tested the effects of *MdWRKY87* overexpression on adventitious root formation in *MdWRKY87*-OE lines #9, #5 and #6 (**Figures 3A**, **B**). During the adventitious rooting process, there were obvious morphological differences between *MdWRKY87*-OE transgenic plants and non-transformed wild type (WT) (**Figure 3A**). The adventitious rooting rate, root number and root length per stem were significantly higher in *MdWRKY87*-OE lines than that in WT plants (**Figures 3C–E** and **Supplemental Movie S1**). These results supported the notion that high *MdWRKY87* expression correlates positively with adventitious rooting.

**Figure 4:**
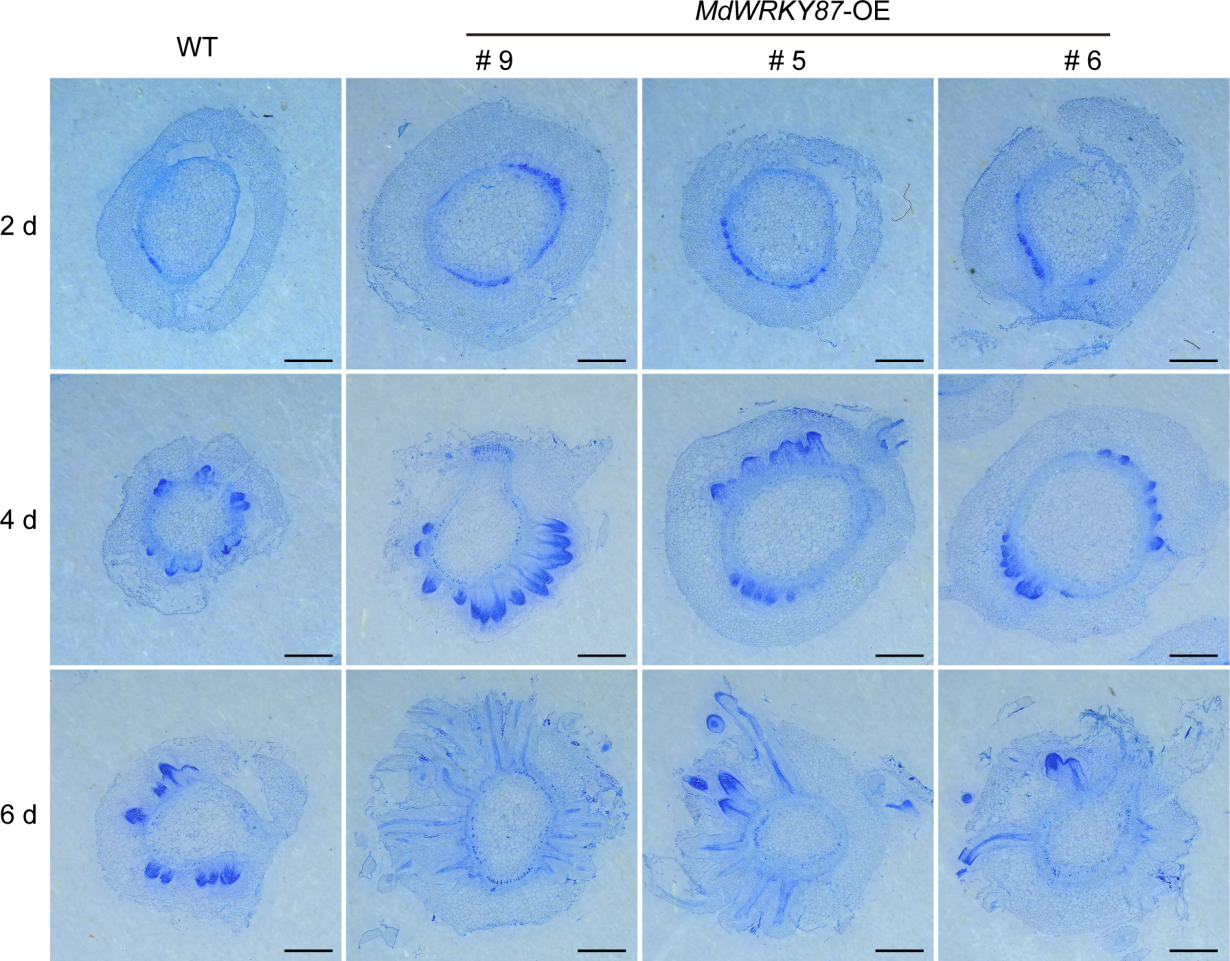
*MdWRKY87* accelerates the formation of adventitious root primordium in transgenic tobacco (*N. tabacum*) plants. Cross-sections of stem bases at 2, 4 and 6 days after transplanting during adventitious root formation in WT and *MdWRKY87-*OE lines. Sections were stained by toluidine blue. Scale bar = 1 mm.

To check whether *MdWRKY87* affects the initiation of adventitious root primordia, we conducted the cross sections of the stems of WT and *MdWRKY87-OE* transgenics lines during adventitious rooting. The initiation of adventitious root primordia was accelerated in *MdWRKY87-OE* transgenics lines from 2 days after subculture on MS (**Figure 4**). Moreover, the adventitious root primordium in *MdWRKY87-OE* transgenics lines were well-developed compared with the WT plantlets at 4 days after subculture, suggesting that high *MdWRKY87* expression accelerates the initiation and development of adventitious root primordia.

### 3.4 Interaction between *MdWRKY87* and auxin during adventitious rooting

**Figure 5:**
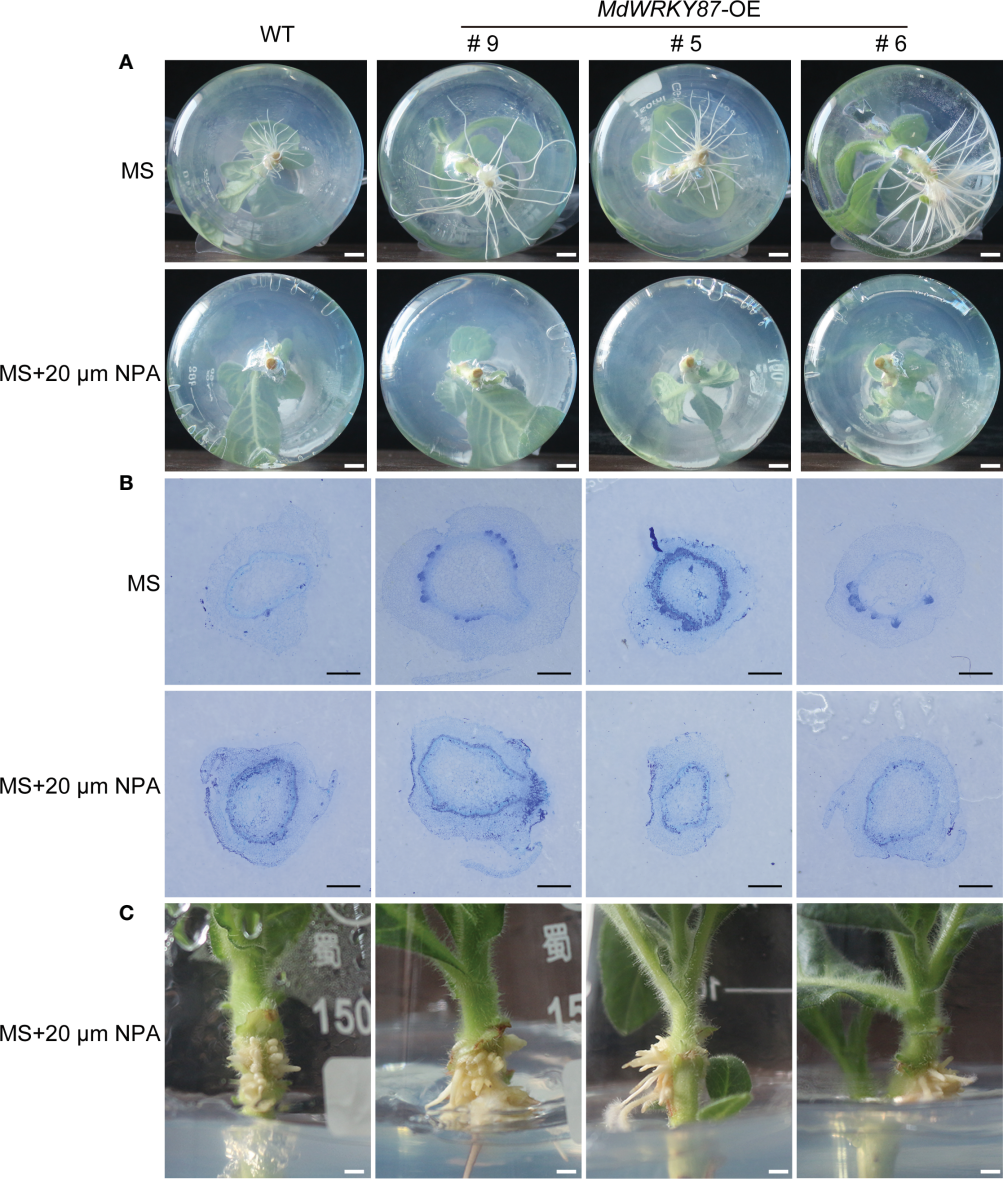
Effects of NPA on adventitious root formation in transgenic tobacco and wild-type plants. **(A)** Phenotypes of 10-day-old plants (WT, and *MdWRKY87-*OE lines) grown on MS medium with 20 µM NPA. **(B)** Cross-sections of stem bases at 5 days after transplanting during adventitious root formation in WT and *MdWRKY87-*OE lines. Sections were stained by toluidine blue. Scale bars = 500 µm. **(C)** Stem air root of wild type and *MdWRKY87-*OE lines at 15 days after transplant to MS medium with 20 µM NPA.

To determine whether *WRKY87* regulates adventitious root formation through modulating auxin polar transport, we examined adventitious rooting capacity in wild-type, *MdWRKY87*-OE tobacco plants stem cuttings grown on MS medium supplemented with 1-N-naphthylphthalamic acid (NPA). Adventitious rooting was almost absolutely inhibited in both wild-type and *MdWRKY87*-OE tobacco plants treated with 20µM NPA (**Figure 5A**). To check whether NPA affects the initiation of adventitious root primordia, we conducted the cross sections of the stems of WT and *MdWRKY87*-OE lines during adventitious rooting. The initiation of adventitious root primordia was both inhibited in wild-type and *MdWRKY87*-OE transgenic lines under NPA treatment (**Figure 5B**). Dense aerial roots developed on the stems of transgenic and wild-type plants under NPA treatment (**Figure 5C**).

### 3.5 Transcriptome profiling of *MdWRKY87*-dependent gene expression during adventitious root formation

**Figure 6:**
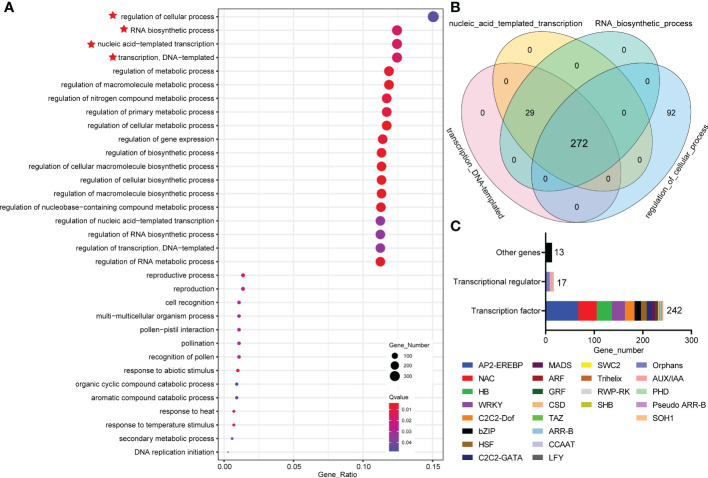
Comparative transcriptome analysis of the stem of WT and *MdWRKY87*-OE tobacco plants. **(A)** Functional categorization of DEGs based on the biological process of Gene Ontology (GO). The symbol red star represent the top 4 GO terms. **(B)** Venn diagram of DEGs in top 4 GO terms based on the biological process of GO. **(C)** The analysis of overlapping DEGs involved in “transcription, DNA-templated”, “nucleic acid-templated transcription”, “RNA biosynthetic process” and “regulation of cellular process”.

To understand how *MdWRKY87* regulates adventitious root development, we conducted a comparative transcriptome analysis of the stem of WT and *MdWRKY87*-OE tobacco plants. A total 6690 differentially expressed genes (DEGs) in tobacco were identified, including 2000 downregulated and 4690 upregulated genes (**Supplemental Figure S2**). These DEGs were then subjected to Gene Ontology (GO) functional classification. Within the three GO categories identified, a greatest number of DEGs was significantly enriched in GO categories ‘biological process’ (**Supplemental Figure S3**). Within the ‘biological process’ GO categories, the top 4 GO terms were “regulation of cellular process”, “RNA biosynthetic process”, “nucleic acid-templated transcription” and “transcription, DNA-templated”, respectively (**Figure 6A**). To further dig out the key genes regulated by *MdWRKY87*, we analyzed these four GO categories. Venn diagram analysis showed that 272 overlapping DEGs were identified in these four GO categories (**Figure 6B**). Most of these overlapping genes were annotated as transcription factor (TF) genes and transcriptional regulator (TR) genes (**Figure 6C**). Among these overlapping DEGs, there were 242 TF genes, 17 TR genes, and 13 other genes (**Figure 6C**). Within these TF genes, *AP-ERFBP*, *NAC*, and *HB* type *TF* genes account for a large proportion (**Figure 6C**, **Supplemental Figure S4**). Most of these TR genes are of the types Orphans and *AUX/IAA* (**Figure 6C**, **Supplemental Figure S4**).

**Figure 7:**
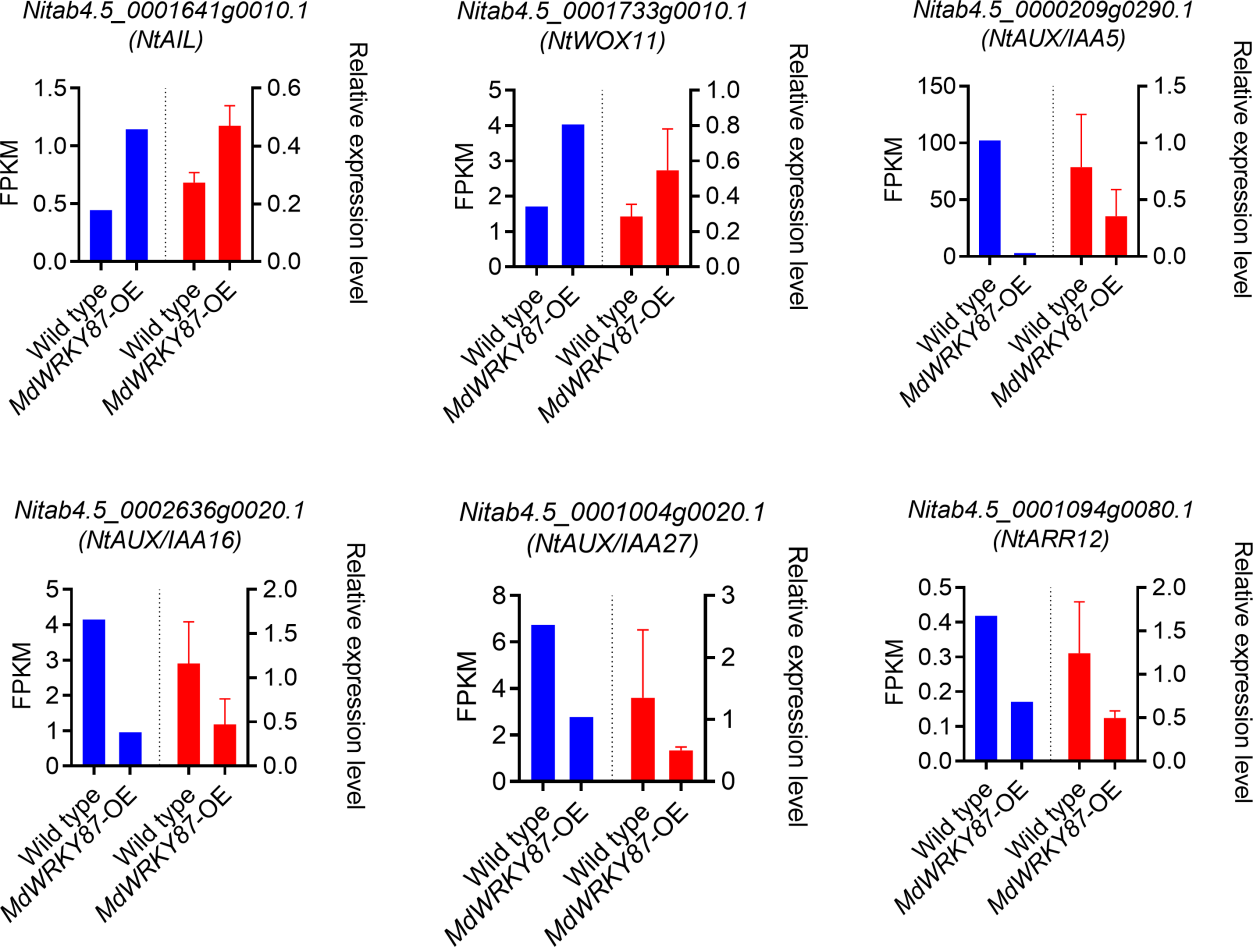
qRT-PCR validation of DEGs in RNA-seq data. Six genes were selected from RNA-seq differentially expressed genes (DEGs) to be validated by qRT-PCR. The data were reported as the means ± SE of three biological replicates. A comparison between the gene expression ratios obtained from RNA-seq data and qRT-PCR.

Further analysis indicated these TF and TR genes mainly enriched in auxin and cytokinin signaling pathway. It is well known auxin and cytokinin appear to play antagonistic roles in the adventitious rooting process. Among these TF and TR genes family, previous study has demonstrated that *AUX/IAAs*, *AINTEGUMENTA* (*ANT*), *AINTEGUMENTA LIKE1* (*AIL*), *WUSCHEL-RELATED HOMEOBOX* (*WOX*), and type-B *cytokinin Response Regulator* (*RR*) family genes involved in adventitious rooting process (Ramírez-Carvajal et al., 2009; Rigal et al., 2012; Lakehal et al., 2019; Geng et al., 2023). In genes up-regulated by *MdWRKY87*, *WUSCHEL-RELATED HOMEOBOX11* (*WOX11*), and *AINTEGUMENTA LIKE1* (*AIL*) are positive regulators of adventitious root formation. For genes down-regulated by *MdWRKY87*, *AUX/IAAs* and type-B *Response Regulator* genes are negative regulator of adventitious root formation. Hence, negative regulation of *AUX/IAAs* and type-B *Response Regulator* genes and positive regulation of *WOX11* and *AIL* by MdWRKY87 contributes to the enhanced adventitious rooting ability in transgenic tobacco plants. The expression level of these genes was confirmed by quantitative real-time (qRT)-PCR, thus supporting the RNA-seq results (**Figure 7**).

## 4 Discussion

Although, it is well known that juvenile or rejuvenated phase leafy cuttings are much easier to root than the adult ones in perennial woody plants, the underlying molecular mechanism that mediates these differences is largely unknown.

### 4.1 Up-regulate *MdWRKY87* contributed to age-dependent adventitious rooting-competent in apple rootstock

For rooting recalcitrant woody plants, juvenility is necessary for efficient adventitious rooting. In general, rooting rates in adult plants are usually lower than in juvenile plants (Xu et al., 2017). Recent studies have provided a paradigm for the molecular basis of age-dependent adventitious rooting ability (Sun and Zhu, 2021). According to previous reports, we can understand the molecular basis from at least two independent signaling pathways: (i) *via* the accumulation of EIN3 protein in adult plants, which directly suppresses expression of *WUSCHEL RELATED HOMEOBOX* (*WOX*) genes to inhibit rooting (Ma et al., 2020); (ii) the miR156-SPLs pathway, which modulates root regeneration by crosstalk with auxin signaling pathway (Xu et al., 2016; Xu et al., 2017; Ye et al., 2020a; Ye et al., 2020b; Li et al., 2021). Whether there is other regulation mechanism controlling age-dependent adventitious rooting ability? Here we demonstrated that higher expression level of *MdWRKY87* contributing to adventitious rooting-competent in juvenile apple rootstock independent of miR156/SPL pathway. As revealed by qRT-PCR, the expression level of *MdWRKY8*7 was positively correlated with adventitious rooting ability (**Figures 1D**, **F**). Over-expressing of *MdWRKY87* in tobacco leads to enhanced adventitious rooting ability, more adventitious root number and accelerated adventitious rooting process (**Figures 3**, **4** and **Supplemental Movie S1**). In addition, *SPL* family genes was not found in the transcriptome data of *MdWRKY87-OE* transgenic plants. It has been widely reported that WRKY transcription factors participate in the regulation of plant growth and development, abiotic stress responses, and disease response. However, the function of WRKY involved in adventitious rooting remains largely unknown. Here, we found WRKY87 transcription factors play essential role in adventitious root formation. In agreement to this result, PuWRKY75 was identified to control the low phosphorus driven adventitious root formation in *Populus ussuriensis* (*Pu*) (Wang et al., 2022). In addition, it was interesting that PuWRKY75 act as a transcriptional enhancer, but MdWRKY87 act as a transcriptional inhibitor (**Figures 2C–E**). There will be more *WRKY* genes, which be identified involving in adventitious rooting process in the future research.

### 4.2 *MdWRKY87* involved in auxin signaling pathway during adventitious rooting

It was well known that auxin play a dominant role in regulation of adventitious root formation (Lakehal et al., 2019). To further identify how *MdWRKY87* regulate adventitious root formation, we analyzed the interaction between *MdWRKY87* and auxin. *MdWRKY87* has significantly enhanced expression levels in response to exogenous IBA treatment (**Figures 1D**, **F**). In addition, *pro MdWRKY87*: *GUS* transiently transformed tobacco leaves exhibited induction of GUS activity after spraying with IBA and the promoter region of *MdWRKY87* has auxin response element (**Figure 1G**). These suggest that *MdWRKY87* acts downstream of auxin to regulate adventitious root formation. However, the adventitious rooting ability was inhibited in *MdWRKY87-*OE transgenic tobacco lines upon treatment with the polar auxin transport inhibitor NPA (**Figure 5**). Taken together, we conclude *MdWRKY87* act not only downstream of auxin, but also feedback regulation during adventitious rooting process. Consistent with this, previous data also demonstrate that WRKY71/EXB1 play pivotal roles in shoot branching by regulating auxin pathways (Guo et al., 2015). However, specific mechanism underlying the crosstalk between MdWRKY87 and auxin signaling needs to be further demonstrated.

### 4.3 *MdWRKY87*-dependent regulation of adventitious rooting related genes in transgenic tobacco

*MdWRKY87* was known to control adventitious root formation (**Figure 3**), but the regulatory role of *MdWRKY87* was unknown. Comparative transcriptome profiling between the wild type and *MdWRKY87-OE* transgenic plants was conducted in this study (**Figure 6**). A set of 272 overlapping DEGs were identified through GO enrichment analysis, implying their potential importance for MdWRKY87-dependent adventitious rooting formation (**Figure 6B**). Among these DEGs, we found *MdWRKY87* overexpression can activate the expression of adventitious rooting-induced genes, such as *WOX11* and *AIL*, and the counterparts of *WOX11* and *AIL* promote adventitious root formation in *A.thaliana* and poplar (Rigal et al., 2012; Hu and Xu, 2016; Geng et al., 2023). In addition, *MdWRKY87* overexpression can inhibit the transcription of adventitious rooting-repressed genes, such as *AUX/IAAs* and *RRs*, and the counterparts of *AUX/IAAs* and type-B *RRs* inhibit adventitious root formation in Arabidopsis, apple, and poplar (Ramírez-Carvajal et al., 2009; Lakehal et al., 2019; Zhao et al., 2020). Similarly, it was demonstrated that PuWRKY75 interacted with PuMYB40 and directly co-regulate *PuLRP1* and *PuERF003* to promote adventitious root formation in *P. ussuriensis* (Wang et al., 2022).

In conclusion, a relatively high expression level of *MdWKRY87* contribute to improving adventitious rooting ability. Based on this potential mechanisms, artificial methods for adventitious rooting ability improving will be created *via* manipulating *MdWRKY87* gene expression, especially for rooting recalcitrant woody perennial species.

## Data availability statement

The datasets presented in this study can be found in online repositories. The names of the repository/repositories and accession number(s) can be found in the article/**Supplementary Material**.

## Author contributions

XX, and ZL conceived and designed the experiment. QT and MX conducted the experiment and data analysis. DW, CW, XW and QC contributed to the data analysis. QT and MX wrote the manuscript. DW, CW, XW and QC drafted the discussion and revised the manuscript. All authors contributed to the article and approved the final version.

## Supplementary material

Supplemental Movie S1 Time-lapse video showing the adventitious rooting of *MdWRKY87-OE* transgenic tobacco plants.
