# Supplementary material for: Integrated transgene and transcriptome reveal the molecular basis of MdWRKY87 positively regulate adventitious rooting in apple rootstock
Source: Front Plant Sci. 2023 Jan 26;14:1136616. doi: 10.3389/fpls.2023.1136616 (PMC9909196; doi:10.3389/fpls.2023.1136616)
Supplement: Supplementary file 3 [file DataSheet_1.docx]

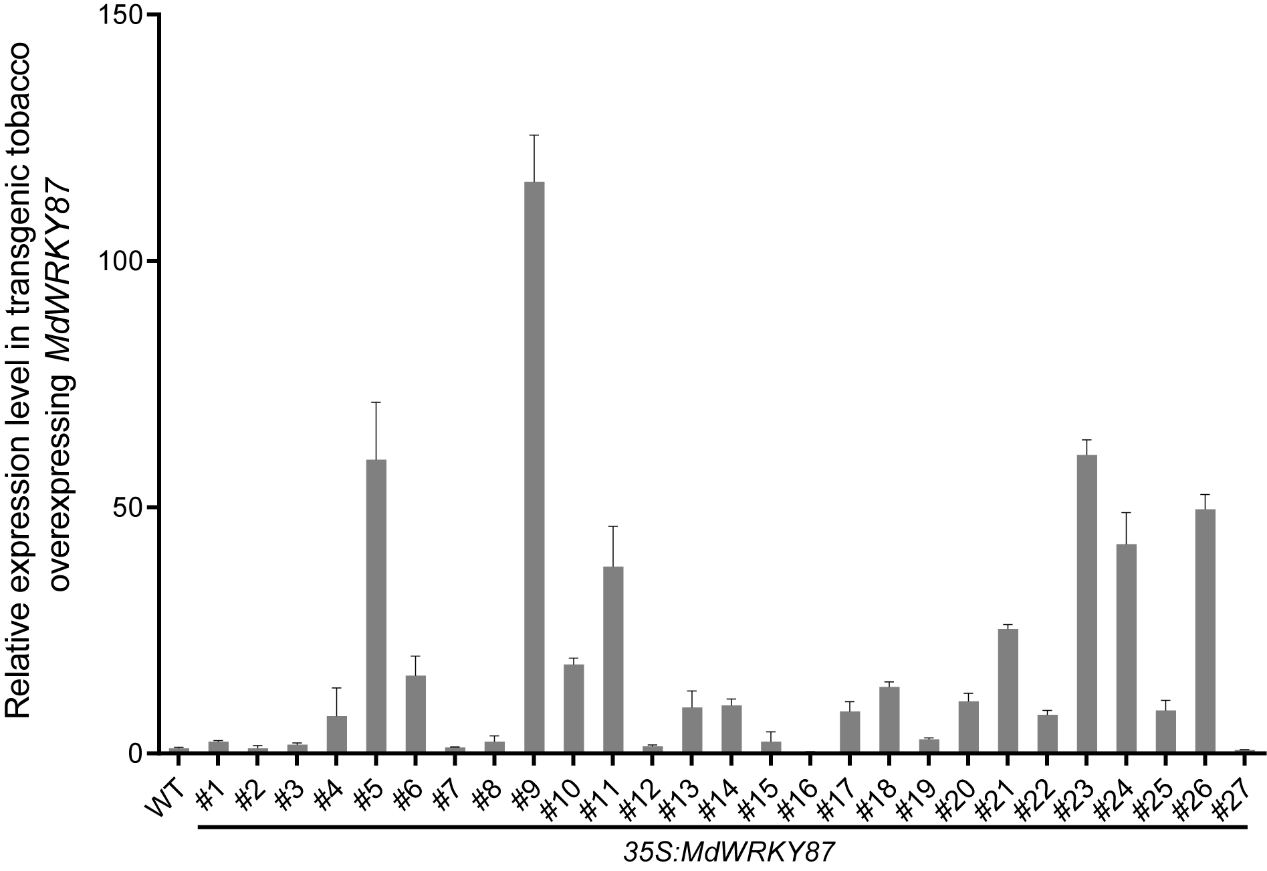


Figure S1. Identification of *MdWRKY87*-OE lines.

Quantitative RT-PCR analysis of *MdWRKY87* expression levels in WT and transgenic lines. The mean values ± SEM are shown for three biological replicates.


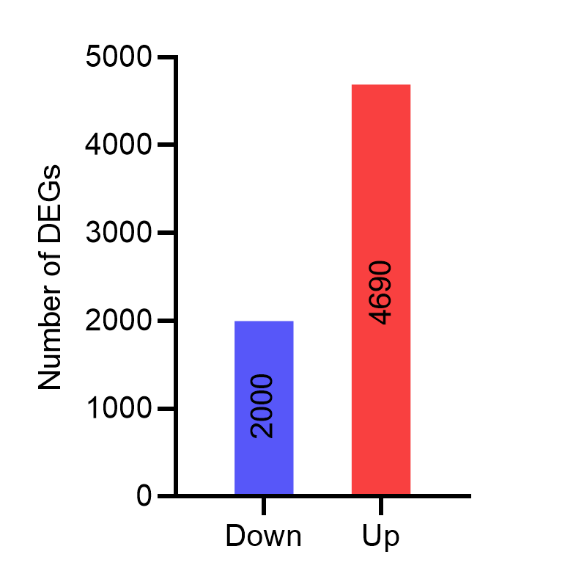


Figure S2. The number of differentially expressed genes (DEGs) in **WT and transgenic lines.**


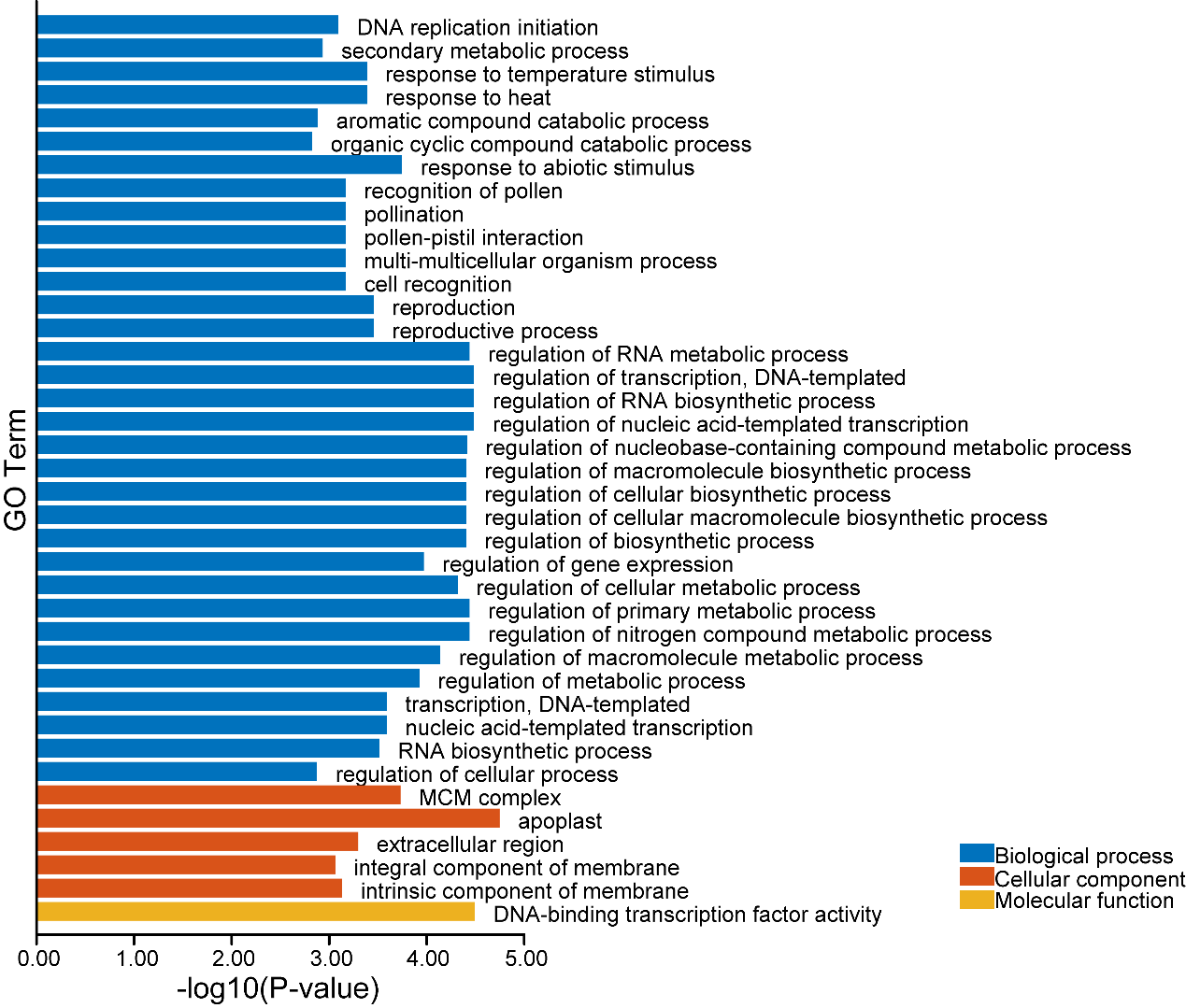


Figure S3. Gene ontology (GO) enrichment analysis for DEGs based on biological process, cellular component and molecular function.


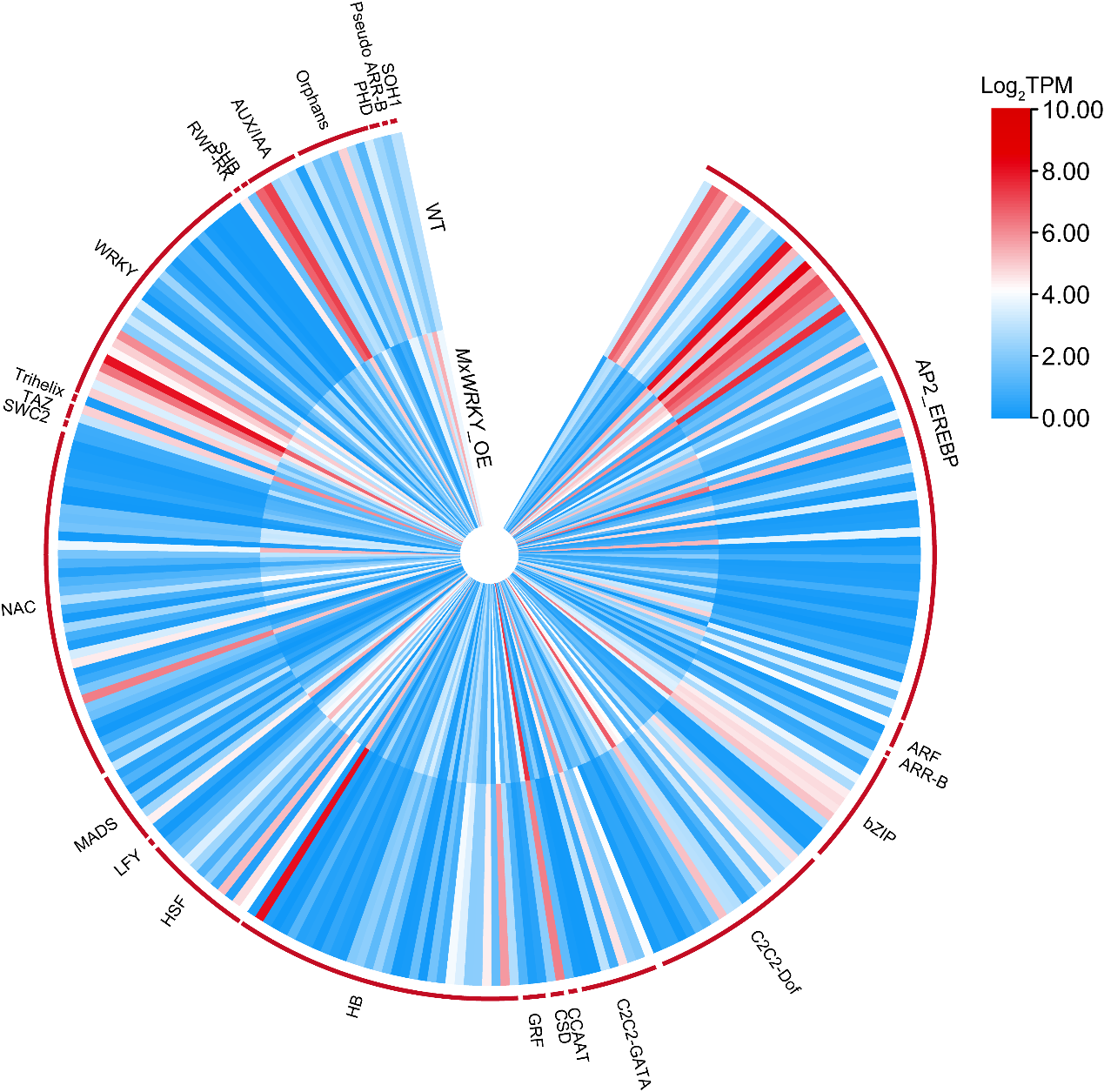


Figure S4. The expression levels of overlapping transcription factor genes and transcriptional regulator genes involved in the top 4 GO terms.
